# Mössbauer and LC-ICP-MS investigation of iron trafficking between vacuoles and mitochondria in *vma2ΔSaccharomyces cerevisiae*

**DOI:** 10.1074/jbc.RA120.015907

**Published:** 2020-12-06

**Authors:** Joshua E. Kim, Shaik Waseem Vali, Trang Q. Nguyen, Andrew Dancis, Paul A. Lindahl

**Affiliations:** 1 Department of Chemistry, Texas A&M University, College Station, Texas, USA; 2 Department of Biochemistry and Biophysics, Texas A&M University, College Station, Texas, USA; 3 Department of Pharmacology, Physiology, and Neuroscience, New Jersey Medical School, Rutgers University, Newark, New Jersey, USA; 4 Division of Hematology-Oncology, Department of Medicine, Perelman School of Medicine, University of Pennsylvania, Philadelphia, Pennsylvania, USA

**Keywords:** V-ATPase, W303, BY4741, EPR, Cup1, homeostasis, iron–sulfur clusters, labile iron pool, labile copper pool, respiratory shield, CD, central quadrupole doublet, FTS, flow-through solution, ISC, iron sulfur cluster, LC-ICP-MS, liquid chromatography interfaced with inductively-coupled-plasma mass spectrometry, LMM, low-molecular-mass, MB, Mössbauer, NHHS, nonheme high spin, ROS, reactive oxygen species, WT, wild type, YPAD, yeast extract peptone adenine dextrose medium

## Abstract

Vacuoles are acidic organelles that store Fe^III^ polyphosphate, participate in iron homeostasis, and have been proposed to deliver iron to mitochondria for iron–sulfur cluster (ISC) and heme biosynthesis. *Vma2Δ* cells have dysfunctional V-ATPases, rendering their vacuoles nonacidic. These cells have mitochondria that are iron-dysregulated, suggesting disruption of a putative vacuole-to-mitochondria iron trafficking pathway. To investigate this potential pathway, we examined the iron content of a *vma2Δ* mutant derived from W303 cells using Mössbauer and EPR spectroscopies and liquid chromatography interfaced with inductively-coupled-plasma mass spectrometry. Relative to WT cells, *vma2Δ* cells contained WT concentrations of iron but nonheme Fe^II^ dominated the iron content of fermenting and respiring *vma2Δ* cells, indicating that the vacuolar Fe^III^ ions present in WT cells had been reduced. However, *vma2Δ* cells synthesized WT levels of ISCs/hemes and had normal aconitase activity. The iron content of *vma2Δ* mitochondria was similar to WT, all suggesting that iron delivery to mitochondria was not disrupted. Chromatograms of cytosolic flow–through solutions exhibited iron species with apparent masses of 600 and 800 Da for WT and *vma2∆*, respectively. Mutant cells contained high copper concentrations and high concentrations of a species assigned to metallothionein, indicating copper dysregulation. *vma2Δ* cells from previously studied strain BY4741 exhibited iron-associated properties more consistent with prior studies, suggesting subtle strain differences. Vacuoles with functional V-ATPases appear unnecessary in W303 cells for iron to enter mitochondria and be used in ISC/heme biosynthesis; thus, there appears to be no direct or dedicated vacuole-to-mitochondria iron trafficking pathway. The *vma2Δ* phenotype may arise from alterations in trafficking of iron directly from cytosol to mitochondria.

Vacuoles are acidic organelles in fungi and plants that are evolutionarily connected to endosomes and lysosomes in humans. Their acidity is essential for sequestering and storing iron and for participating in cellular iron homeostasis (1, 2). The vacuolar ATPase (V-ATPase) is a membrane-bound multisubunit complex that pumps protons from the cytosol into the vacuole. This results in an acidic vacuolar lumen (pH 5.6–6.1) and a slightly basic cytosol (pH ∼ 7.4) (3, 4, 5, 6, 7). *vma*-mutant cells lack functional V-ATPases and grow well only in media buffered between pH 3 and 6, a narrower and more acidic range than is normal for WT cells (7, 8, 9). The pH of the growth medium influences the pH of vacuoles (8). When *vma*-mutant cells were grown at pH 5.5, their vacuolar pH was 5.9 (4, 6, 7). When the same cells were transferred to pH 7.5 medium, vacuolar pH shifted to 7.05. In contrast, vacuoles of WT cells treated equivalently remained at pH 5.9.

V-ATPases consist of two subcomplexes including a peripheral membrane subcomplex V_1_ and an integral membrane complex V_o_. In yeast, V-ATPases are found in vacuolar membranes or vacuole–mitochondria contact sites. Inactivation or deletion of any component subunit leads to a similar mutant phenotype characterized by reduced replicative lifespan, decreased inorganic polyphosphates, sensitivity to oxidative stress, and iron dysregulation. *VMA2* encodes subunit B of the V_1_ subcomplex; the knockout is viable although it lacks V-ATPase activity.

Iron-replete WT vacuoles contain high (mM) concentrations of polyphosphate that coordinate Fe^III^ ions (10, 11, 12). Polyphosphate chains are synthesized by the VTC complex on the vacuolar membrane (13, 14), a process that depends on the proton gradient generated by V-ATPase. Without this gradient, *vma* mutants are deficient in polyphosphate ions and are less able to store iron (15, 16). Thus, polyphosphate ions and protons both impact vacuolar iron metabolism.

Iron homeostasis in yeast involves about two dozen genes called the *iron regulon* (17). These genes are activated when mitochondrial iron–sulfur cluster (ISC) assembly is compromised (18). For example, this condition is evident in strains that are deficient in yeast frataxin homolog 1 (Yfh1) (19), yeast adrenodoxin homolog 1 (Yah1) (20), and the inner membrane transporter Atm1 (21). These cells respond to iron regulon activation by importing excess iron and mobilizing vacuolar iron stores.

The iron regulon is activated in *vma2Δ* cells grown in iron-replete (μM range) media even though their mitochondria are not deficient in proteins involved in ISC assembly (22, 23). This implies that mitochondria cannot assemble sufficient ISCs when the vacuolar lumen is insufficiently acidic, and it highlights an intriguing connection between the two organelles. *vma2Δ* cells have reduced activities of mitochondrial ISC-containing proteins that are involved in respiration, including aconitase, succinate dehydrogenase, and the Rieske iron–sulfur protein (23). *vma*-mutant cells grow poorly on respiring media (3, 22, 24, 25). However, supplementing growth media with mM concentrations of iron a) rescues this phenotype, b) deactivates the iron regulon (22, 23), c) increases the activity of ISC-containing enzymes, d) increases the rate of O_2_ consumption, and e) stimulates respiration (24, 25, 26).

Excess supplemented iron provides the additional iron needed for the biosynthesis of iron-rich mitochondria which are in turn needed for respiration. This requirement is uncompromising, even in WT cells, such that cells grown on iron-deficient minimal medium (MM) succumb to autophagy so that they can recycle sufficient cellular iron for use in mitochondriogenesis (27). During autophagy, Smf3 and Fet5/Fth1 iron exporters on the vacuolar membrane pump iron into the cytosol to satisfy mitochondrial demand. Iron-deficient *vma* mutants have difficulty undergoing autophagy which hinders respiration (27).

When the iron regulon is activated in ISC-mutant cells, the rate of cellular iron import increases dramatically owing to expression of a high-affinity iron importer (composed of Fet3 and Ftr1) on the plasma membrane. Expression of the high-affinity vacuolar iron exporter (composed of Fet5 and Fth1) also increases. Fet3 and Fet5 are paralogous multicopper oxidases controlled by the iron regulon. As a result, the vacuoles of ISC-mutant cells are nearly devoid of iron, and much of the excess iron that flows into the cytosol ultimately accumulates in mitochondria as Fe^III^ oxyhydroxide phosphate–associated nanoparticles (19, 20, 21).

*vma*-mutant cells have difficulty importing extracellular iron because copper-containing Fet3 has difficulty maturing (*i.e.*, metallating with copper) in the Golgi (1, 28). Whether these cells also have difficulty exporting vacuolar iron is unknown, but this seems likely, given the similarities between Fet3 and Fet5. In any event, the concentration of iron in *vma*-mutant cells is not excessive relative to WT cells even though the iron regulon is activated. *vma2Δ* cells reportedly contain only ∼30% more iron than WT cells (23). In contrast, ISC mutants contain an order-of-magnitude more iron than WT cells.

The mechanism by which a decline in the acidity of vacuoles causes a decline of mitochondrial function, and how excessive iron rescues this decline, has been investigated by Kane (23) and more recently by Hughes (26). Both hypothesize that the flow of iron from vacuoles to mitochondria is disrupted in *vma*-mutant cells and that excess media iron re-establishes this pathway. Besides sequestering iron, vacuoles store amino acids, including cysteine (29). Hughes *et al.* (26) found that loss of vacuolar acidity increases nonvacuolar pools of cysteine and other amino acids. One intriguing possibility is that excess cysteine in the cytosol binds iron, thereby blocking import into mitochondria.

*vma* mutants generate high levels of reactive oxygen species (ROS) and are hypersensitive to oxidative stress (5, 22, 30, 31). They are more easily intoxicated by high levels of transition metals than WT cells. This likely occurs because *vma* cells have difficulty storing metals in their vacuoles, which otherwise imparts metal resistance to the cell (17, 22, 24, 32, 33). Surprisingly, mitochondrial respiration is *not* the major source of ROS in *vma2Δ* cells (22). Rather, excessive ROS is likely associated with iron dyshomeostasis and increased Fenton chemistry (5, 23).

Also surprising is that the relationship between vacuolar acidity, cellular iron, mitochondrial function, and ROS damage is related to human aging and longevity. Hughes and Gottschling found that a decline of vacuolar acidity is associated with age-related deterioration of mitochondria (34). Overexpressing V-ATPase subunits suppresses mitochondrial dysfunction in aging cells by hyperacidifying vacuoles. Young yeast cells treated with a V-ATPase inhibitor and mutant cells lacking V-ATPase subunits both have impaired mitochondrial morphology similar to the organelle in aged cells (34).

The objective of the current study was to better understand the connection between vacuolar acidity, mitochondrial function, and iron homeostasis by using powerful biophysical and bioanalytical probes, namely Mössbauer (MB) and EPR spectroscopies and liquid chromatography interfaced with inductively-coupled-plasma mass spectrometry (LC-ICP-MS). Mitochondria, vacuoles, and the cytosol of *vma2Δ* cells were isolated and investigated. Our results confirm and extend some but not all relevant previous results, with differences probably due to the background strains and media/conditions used. New insights into the connection between vacuoles and mitochondria are presented, highlighting the potential importance of low-molecular-mass (LMM) iron, copper, and sulfur species in the cytosol of these yeast cells.

## Results

A haploid *VMA2* deletion strain was generated from a W303 parent to allow for direct comparison to previous biophysical studies that used the same genetic background (10, 11, 20, 21, 35, 36, 37, 38, 39). We refer to W303 and the mutant strain as WT and *vma2Δ*_*W*_, respectively. We initially investigated whether the iron content of these two strains differed and whether the pH of the medium influenced that content. WT and *vma2Δ*_*W*_ cells were grown in MM containing 2% glucose as the carbon source (fermentation) and supplemented with iron (typically 40 μM ^57^Fe^III^ citrate). Medium pH was buffered using citric acid and sodium citrate. Under these conditions, *vma2Δ*_*W*_ cells grew well at pH values 3, 4, and 5 although growth was inhibited when the medium pH was 6 or 7; in contrast, WT cells grew well in media buffered from pH 3 to 7.

Whole-cell iron concentrations (Table 1) revealed no obvious pH dependence; however, some strain-dependent trends were evident. *vma2Δ*_*W*_ cells contained 30% less iron, 60% less phosphorus, 6× less manganese, 4× less zinc, and 2.5× more copper than WT cells. The increased copper when considered with previous reports that Fet3 in *vma2Δ* cells is not metallated in the Golgi (1, 28) suggested that copper was dysregulated in these mutant cells and that *vma2Δ* cells compensated by importing excessive copper.

**Table 1:** Metal concentrations in WT and *Vma2Δ*_*W*_ whole cells and cytosol fractions

Sample	n	Fe	Cu	Mn	Zn	P
WT pH 7	3	200 ± 30	100 ± 40	27 ± 8	500 ± 200	190 ± 10
WT pH 6	4	320 ± 40	100 ± 100	31 ± 3	1300 ± 500	500 ± 100
WT pH 5	4	300 ± 40	78 ± 5	60 ± 20	900 ± 100	500 ± 100
WT pH 4	3	260 ± 20	150 ± 10	44 ± 2	470 ± 60	280 ± 20
WT pH 3	4	190 ± 40	130 ± 10	18 ± 6	840 ± 40	210 ± 30
*vma2Δ*_*w*_ pH 5	4	250 ± 40	240 ± 50	10 ± 1	193 ± 6	170 ± 40
*vma2Δ*_*w*_ pH 4	2	128 ± 3	220 ± 20	7 ± 4	130 ± 20	67 ± 9
*vma2Δ*_*w*_ pH 3	5	160 ± 40	340 ± 40	2 ± 1	230 ± 20	104 ± 7
WT cytosol	2	270 ± 50	100 ± 7	25 ± 1	800 ± 100	490 ± 40
*vma2Δ*_*w*_ cytosol	4	200 ± 70	280 ± 70	8 ± 2	250 ± 80	170 ± 10
WT cytosol (1 μM Fe)	2	230 ± 20	130 ± 20	11 ± 3	340 ± 40	300 ± 30
WT FTS (1 μM Fe)	2	66 ± 2	13 ± 4	7 ± 2	130 ± 20	170 ± 6

Cells were grown on MM supplemented with 40 μM iron citrate (unless specified otherwise) and 10 μM CuSO_4_. Samples were whole cells unless otherwise indicated. n, number of independent replicates. Concentrations are in μM except for P which is in mM. Reported cytosol and FTS concentrations are measured values after being multiplied by a 14× dilution factor.

FTS, flow-through solution; MM, minimal medium.

**Figure 1:**
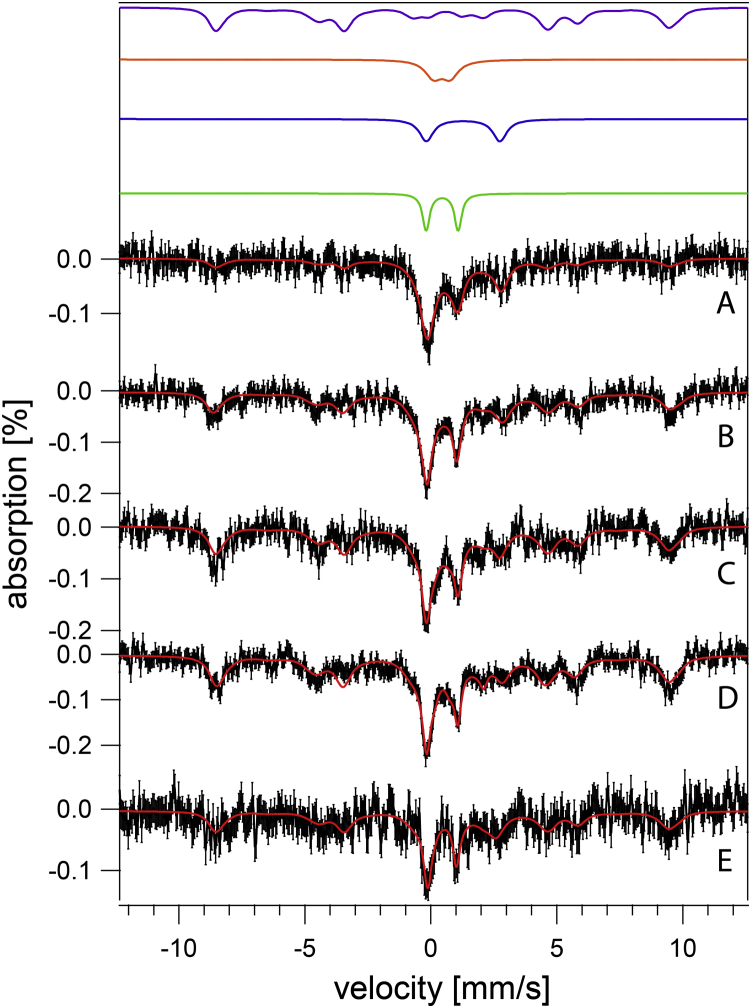
**Mössbauer spectra of fermenting WT cells at varying pH (MM supplemented with 40 μM**^**57**^**Fe**^**III**^**citrate and 10 μM CuSO**_**4**_**).***A*, pH 7, *B*, pH 6, *C*, pH 5, *D*, pH 4, *E*, pH 3. Data are *black hashmarks*; composite simulations are the *solid red lines*. The *green line* simulates low-spin Fe^II^ hemes and [Fe_4_S_4_]^2+^ clusters. The *blue line* simulates nonheme high-spin Fe^II^. The *purple line* simulates nonheme high-spin Fe^III^. Individual simulations were combined to yield the composite simulation of of the WT pH 5 spectrum. MM, minimal medium.

MB spectra of both strains were collected to investigate the type of iron present. WT cells exhibited qualitatively similar spectra regardless of pH (Fig. 1), but some subtle pH-dependent trends were evident. At low pH, over 60% of spectral intensity was a magnetic feature originating from nonheme high-spin (NHHS) Fe^III^. The solid purple line in Figure 1 is a simulation using parameters in Table S1. The Fe^III^ ions are coordinated to anionic polyphosphate chains in vacuoles (10, 11). With increasing pH, the percentage of cellular iron in this form declined to ∼30% at pH 7. We conclude that WT vacuoles store less iron as medium pH increases.

Also evident in WT MB spectra was the central doublet (CD) with isomer shift and quadrupole splitting parameters typical of [Fe_4_S_4_]^2+^ clusters and low-spin Fe^II^ hemes (simulated by the green line in Fig. 1). MB spectra of isolated mitochondria are dominated by the CD (36, 37, 38), which originates mainly from iron-rich respiratory complexes and respiration-related iron-containing proteins, *e.g.*, aconitase. A substantial portion of the CD intensity in whole cells is due to cytosolic [Fe_4_S_4_]^2+^-containing proteins. Percentagewise, the spectral intensity of the CD in whole-cell WT spectra increased from ∼15% at low pH values to ∼30% at pH 7. This increase likely arose from the percentagewise decrease of vacuolar iron, such that the absolute concentration of the [Fe_4_S_4_]^2+^ clusters and LS Fe^II^ hemes that give rise to this spectral feature were fairly constant with changes in pH.

Another ∼20% of the intensity of WT cell spectra was a quadrupole doublet (simulated by the blue line in Fig. 1) with parameters typical of NHHS Fe^II^ complexes coordinated by 5 to 6 O/N donors (δ = 1.27 mm/s; ΔE_Q_ = 2.9 mm/s). Such species are probably located in the cytosol and mitochondria, among other regions of the cell. Another ∼10% of spectral intensity consisted of poorly resolved material near the center of the spectrum (at ∼ 0 velocity). In Figure 1, this material was simulated (orange line) assuming parameters of Fe^III^ phosphate–associated nanoparticles. Apart from the modest decline of vacuolar iron with increased pH, the iron in WT cells generally remained invariant over four orders-of-magnitude of acidity in the growth media. This implies that intracellular pH of WT cells is well regulated against extracellular perturbations, probably due to the activities of proton pumps on the plasma and vacuolar membranes.

**Figure 2:**
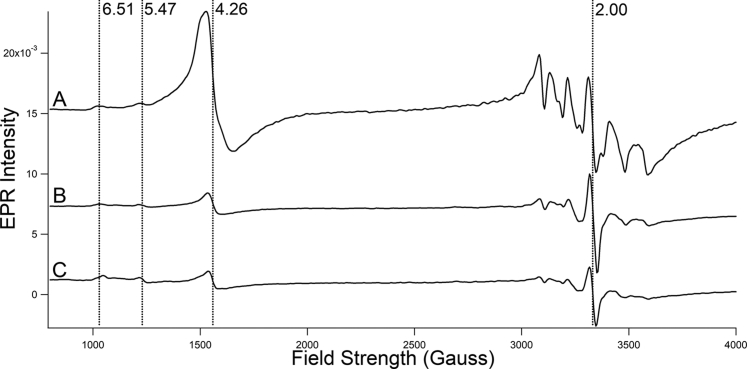
**EPR spectra of fermenting WT and *vma2Δ***_***W***_**whole cells.***A*, WT; *B*–*C*, independent replicates of *vma2Δ*_*w*_. Spectra were collected at 10 K; other conditions are given in Experimental procedures.

The WT EPR spectrum of whole WT cells cultured at pH 5 with 40 μM supplemented Fe^III^ citrate (Fig. 2*A*) exhibited signals similar to those observed previously (11, 38). Vacuolar Fe^III^ exhibited a prominent g = 4.3 signal. Features at g = 6.5 and 5.5 are typical of high-spin Fe^III^ hemes. A hyperfine-split signal arising from Mn^II^ ions was observed at g = 2.00, as was an isotropic radical signal. No signals attributable to Cu^II^ ions were observed.

**Figure 3:**
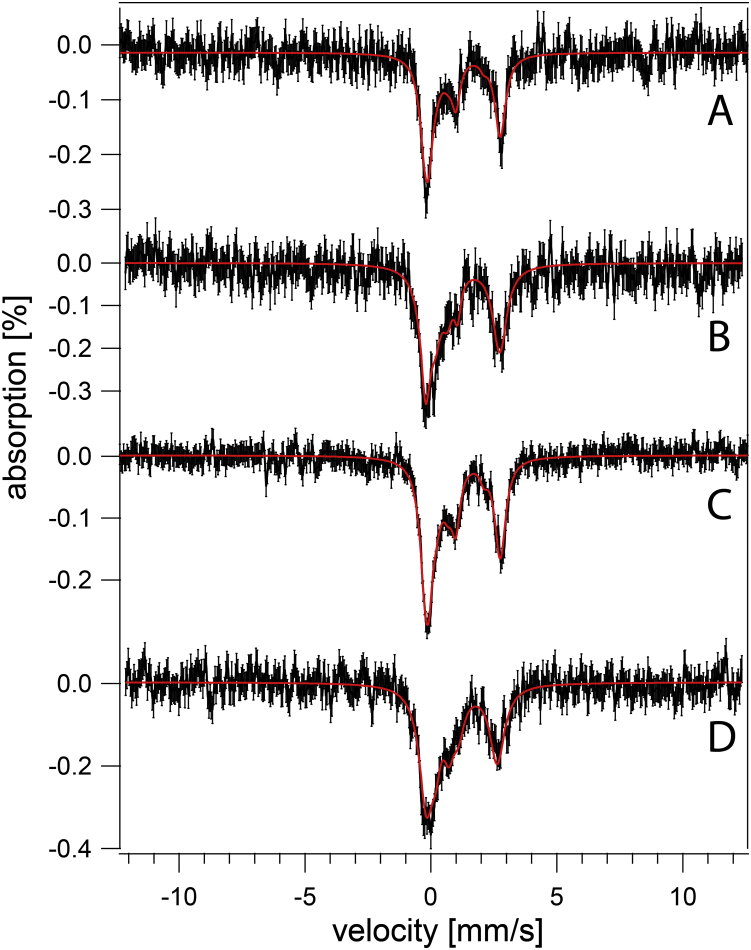
**Mössbauer spectra of *vma2Δ* cells at varying pH (MM supplemented with 40 μM ferric citrate and 10 μM copper sulfate).***A*, pH 5, *B*, pH 5 with 1 mM supplemented cysteine, *C*, pH 4, *D*, pH 3. *Black*, collected spectrum. *Red*, composite simulation. MM, minimal medium.

MB spectra of *vma2Δ*_*W*_ cells grown in MM at pH 3, 4, and 5 were dominated by an Fe^II^ quadrupole doublet (Fig. 3) that accounted for ∼60% of spectral intensity. The high-energy line of the CD was also evident as a partially resolved shoulder on the low-energy line of the dominating Fe^II^ doublet at ∼ + 1 mm/s. Like WT cells, the iron content of *vma2Δ*_*W*_ cells was generally invariant in media in which acidity varied by three orders of magnitude. We had predicted that the magnetic feature due to vacuolar Fe^III^ in MB spectra of WT cells would also be present in spectra of *vma2Δ*_*W*_ cells grown at pH 3 or 4, given that the acidity of vacuoles lacking functional V-ATPase is affected by the pH of the growth media. This prediction was not realized, possibly for reasons discussed below.

The CD represented ∼20% of the spectral intensity for *vma2Δ*_*W*_ cells at each pH within this range. Assuming an average cellular iron concentration of 180 μM (Table 1) suggests that ∼40 μM iron in these cells arose from [Fe_4_S_4_]^2+^ clusters and LS Fe^II^ hemes. For WT cells, with an average iron concentration of ∼250 μM and ∼20% CD intensity, the corresponding concentration would be ∼50 μM. The two concentrations are the same within our uncertainties. We had expected that the concentration of such centers in *vma2Δ*_*W*_ cells would have been much lower and that most of the iron in these cells would have been Fe^III^ phosphate–associated nanoparticles as is observed in other ISC-mutant cells in which the iron regulon is activated. However, MB spectra of *vma2Δ*_*W*_ cells did not exhibit intense features typical of nanoparticles nor did they provide clear evidence that the iron regulon was activated.

EPR spectra of *vma2Δ*_*W*_ cells at pH 5 (Fig. 2, *B*–*C*) supported the MB spectra and provided new insights. The intensity of the g = 4.3 signal due to vacuolar Fe^III^ was strongly reduced relative to in WT spectra, consistent with the absence of the magnetic feature due to vacuolar Fe^III^ in corresponding MB spectra. Minor features attributed to high-spin hemes at g = 6.5 and 5.5 were comparable in intensity to those of WT, suggesting normal levels of cellular hemes in the mutant cells. The hyperfine-split signal due to Mn^II^ ions was dramatically reduced in *vma2Δ*_*W*_ EPR spectra, consistent with the 6-fold decline of Mn concentration relative to in WT cells (Table 1). No signals attributable to S = ½ Cu^II^ ions were observed, despite the 2.5-fold increase in Cu concentration in the mutant cells. This suggests that the additional Cu ions in *vma2Δ*_*W*_ cells were in the reduced diamagnetic Cu^I^ state.

We assessed whether the dominating Fe^II^ species in *vma2Δ*_*W*_ cells arose from an accumulation of LMM iron complexes in the cytosol. The cytosol was isolated from WT and *vma2Δ*_*W*_ cells, and the concentration of iron in these cytosol isolates was determined (Table 1). The iron concentration of cytosol from both strains was high (200, 270 μM), similar to values reported previously (38). Cytosol solutions were passed through a 10-kDa cutoff membrane, and the metal contents of flow-through solutions (FTSs) were also determined. We previously found that LMM iron species constituted ca. 70% of cytosolic iron at 40 μM iron supplementation (38). Here, we observed that ∼30% of cytosolic iron from cells grown with 1 μM Fe was present in an LMM form (Table 1). The concentration of LMM iron in the cytosol was higher than expected by an order-of-magnitude. We suspected that iron-rich vacuoles may have burst during cytosol isolation and metals from the ruptured organelles leached into the cytosol. However, vacuoles contain little iron at 1 μM iron supplementation (37, 38), yet the iron concentrations of cytosol isolated from 1 μM and 40 μM iron-supplemented WT cells were similar. Thus, vacuole-bursting seems unable to explain the high concentrations.

**Figure 4:**
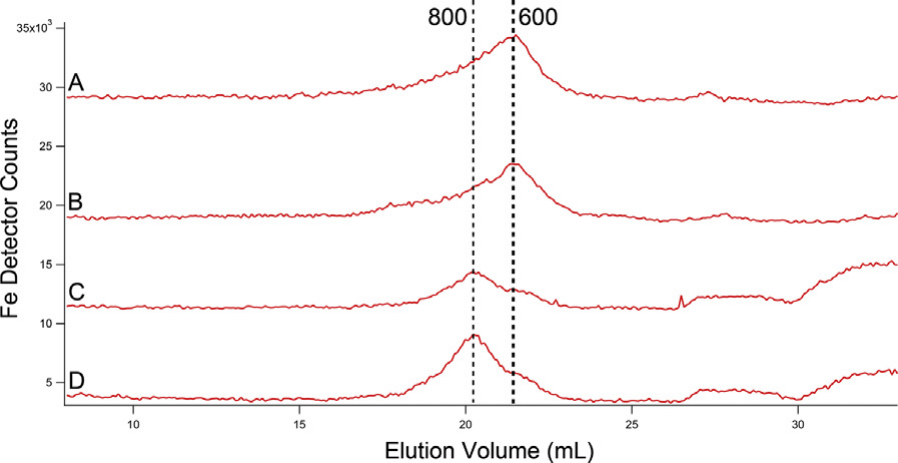
**Iron-detected chromatogram of isolated cytosol FTS.***A*–*B*, two independent WT batches; *C*–*D*, two independent *vma2Δ*_*W*_ batches. WT chromatograms for Fe and elements in subsequent figures were from (38). FTS, flow-through solution.

FTSs were subjected to LC-ICP-MS chromatography. The resulting traces (Fig. 4) revealed unresolved LMM iron-detected peaks for each strain. The dominant LMM iron species in WT cytosol FTS migrated with an apparent mass of ∼600 Da (all masses quoted in the text are apparent), whereas the dominant LMM iron species in *vma2Δ*_*W*_ cytosol FTS migrated with a mass of ∼800 Da. Both species were present in the FTS of both strains, and their collective intensities were similar. We conclude that the LMM iron in the cytosolic FTS of *vma2Δ*_*W*_ and WT cells are similar though not identical in terms of species and concentration. We caution readers that about half of the LMM iron in the cytosolic fraction adsorbed onto the column.

**Figure 5:**
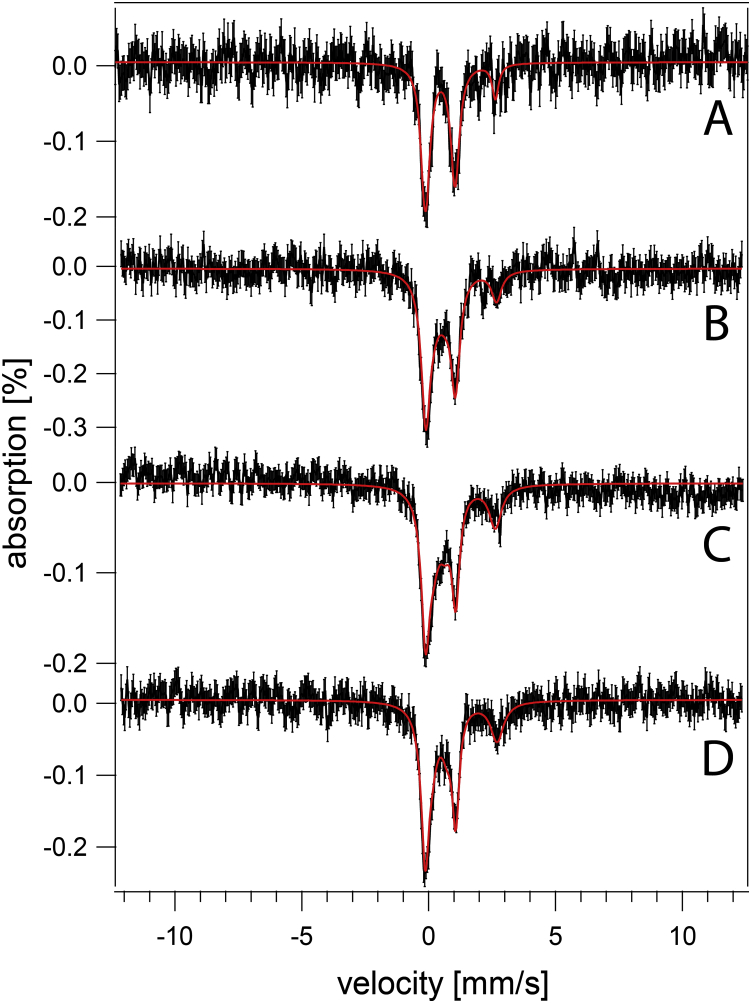
**Mössbauer spectra of mitochondria isolated from two independent batches of fermenting cells (MM supplemented with 40 μM ferric citrate and 10 μM copper sulfate).***A–B*, from WT cells; *C–D*, from *vma2Δ*_*W*_ cells. MM, minimal medium.

We considered that much of the NHHS Fe^II^ in *vma2Δ*_*W*_ cells was located in the mitochondria, and so this organelle was isolated from both WT and *vma2Δ*_*W*_ strains (grown on fermenting MM at pH 5). MB spectra of isolated mitochondria from each strain (Fig. 5) were dominated by the CD (ca. 80% intensity). A minor NHHS Fe^II^ doublet (15%–20%) was also evident. Aconitase activities in these mitochondrial lysates were similar (WT = 2.6 ± 1.2 units/mg protein, *vma2Δ*_*W*_ = 2.3 ± 1.2 units/mg protein; n = 2). Little if any Fe^III^ oxyhydroxide nanoparticles were present in *vma2Δ*_*W*_ mitochondria, unlike mitochondria from strains in which ISC assembly is defective. In summary, the iron contents of mitochondria isolated from WT and *vma2Δ*_*W*_ cells, as evaluated by MB spectroscopy and by the activity of an ISC-containing enzyme, were similar. The low-intensity NHHS Fe^II^ doublet observed in WT and *vma2Δ*_*W*_ mitochondria indicate that mitochondrial Fe^II^ alone cannot account for the dominating Fe^II^ intensities observed in *vma2Δ*_*W*_ whole-cell MB spectra.

**Figure 6:**
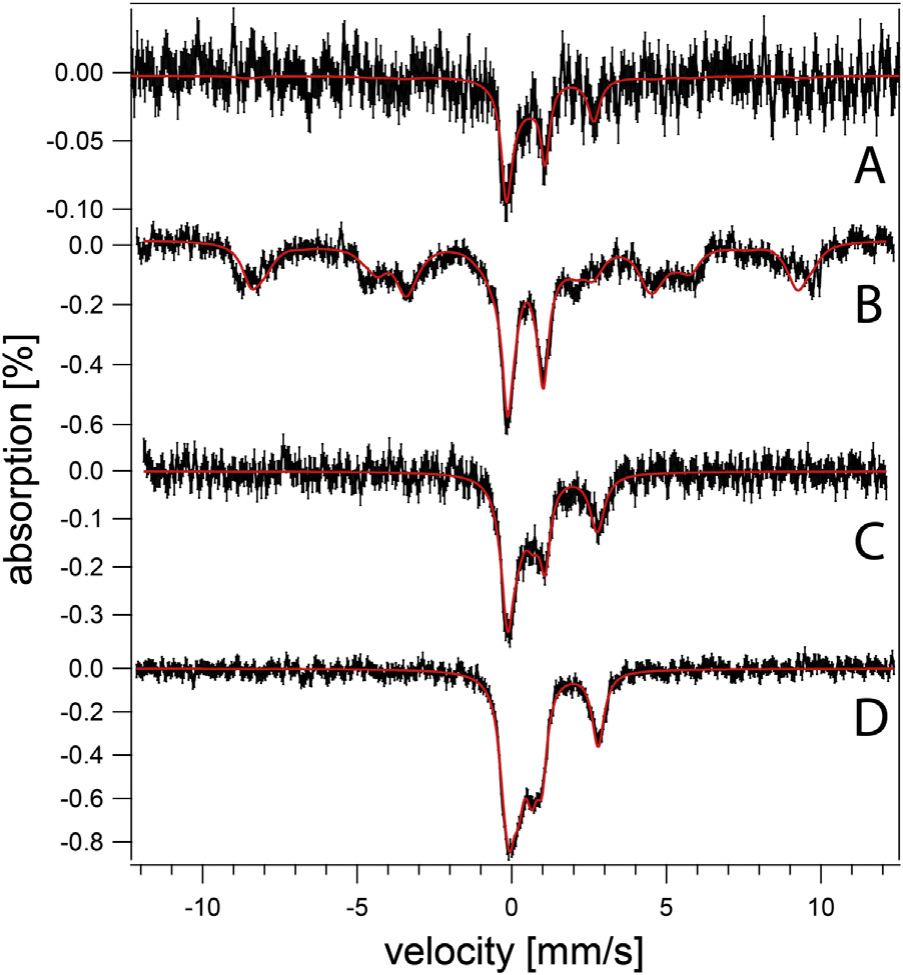
**Mössbauer spectra of respiring WT (*A*–*B*) and *vma2Δ***_***W***_**(*C*–*D*) cells grown on media supplemented with 1 (*A* and *C*) and 40 (*B* and *D*) μM**^**57**^**Fe**^**III**^**citrate and 10 μM copper sulfate and at pH 5.**

We wondered whether the metabolic state of *vma2Δ*_*W*_ cells (fermenting *versus* respiring) affected the iron content of the cells. To address this, WT and *vma2Δ*_*W*_ cells were grown on a respiring carbon source (glycerol/ethanol) in MM buffered at pH 5 and supplemented with 40 μM ^57^Fe^III^ citrate. In this case, *vma2Δ*_*W*_ cells required prior growth in glucose-based media before switching to respiring MM. About 65% of the intensity of the resulting MB spectrum of WT cells (Fig. 6*B*) arose from vacuolar high-spin Fe^III^ (10, 11); another ca. 25% arose from the CD. The higher intensity observed for the CD confirms that respiration-related ISC proteins are upregulated during respiration (36). In contrast, the MB spectrum of respiring *vma2Δ*_*W*_ cells (Fig. 6*D*) lacked the magnetic feature arising from vacuolar Fe^III^ but exhibited an Fe^II^ doublet representing ∼40% of spectral intensity. Over half of the spectral intensity arose from the CD combined with unresolved iron in the middle of the spectrum which fit to a doublet with the parameters of nanoparticles. The cellular concentrations of [Fe_4_S_4_]- and LS Fe^II^ heme-containing species in respiring WT and *vma2Δ*_*W*_ cells were again similar, as observed for fermenting cells. In summary, the iron content of respiring *vma2Δ*_*W*_ cells was similar to that of WT cells, though with a dominance of Fe^II^ instead of vacuolar high-spin Fe^III^. The simplest interpretation is that vacuolar Fe^III^ in WT cells are converted to NHHS mononuclear Fe^II^ and Fe^III^ nanoparticles in fermenting and respiring *vma2Δ*_*W*_ cells.

We considered that some of the dominant NHHS Fe^II^ species in iron-replete *vma2Δ*_*W*_ cells were present in vacuoles, and so two batches of vacuoles from *vma2Δ*_*W*_ cells were isolated and FTSs were prepared. LC traces of these solutions exhibited LMM iron peaks that were either of similar intensity as peaks from WT vacuoles – or less intense (data not shown). However, these studies used a column that was not treated such that adsorption of iron was considerable. We considered that *vma2Δ*_*W*_ vacuoles contained additional iron that adsorbed onto the column, but the iron concentration of those vacuoles was not unusually high.

Cells grown in media containing low concentrations of iron do not store much of the metal in their vacuoles. Thus, the contribution of vacuolar iron to MB spectra can be largely eliminated by growing cells under these conditions. The MB spectrum of respiring WT cells grown in MM supplemented with 1 μM ^57^Fe citrate (Fig. 6*A*) was noisy because the cells contained only 180 μM Fe. However, the S/N was sufficient to conclude that the vacuolar Fe^III^ feature was essentially absent (<15% of spectral intensity). The corresponding spectrum of *vma2Δ*_*W*_ cells, containing 220 μM Fe (Fig. 6*C*), also lacked the vacuolar Fe^III^ feature and also exhibited an intense CD (even stronger than for WT cells). This indicates that *vma2Δ*_*W*_ mitochondria were fully able to generate [Fe_4_S_4_] clusters (and LS Fe^II^ hemes). We conclude that *vma2Δ*_*W*_ mitochondria were able to import cytosolic iron and were able to use it for ISC assembly even though vacuoles were largely devoid of iron. This behavior was unexpected, given previous results which indicated a strong need for functional acidic vacuoles to deliver iron to mitochondria during mitochondriogenesis associated with respiration, as well as with the reported rescue of growth of *vma*-mutant cells with high (mM) concentrations of nutrient iron (22, 24, 26). In summary, these experiments performed with only 1 μM added iron gave essentially the same result in *vma2Δ* cells, showing a normal ISC pool in cells where vacuoles did not store significant iron.

**Figure 7:**
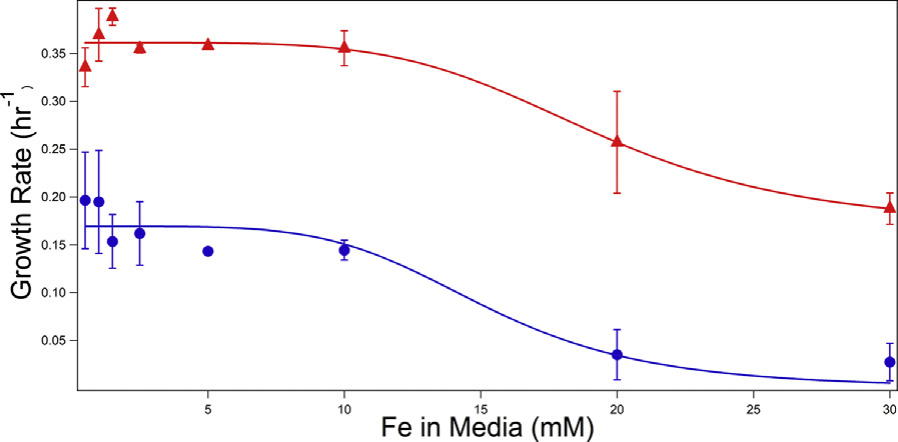
**Plot of the exponential growth rate α of fermenting WT (*red triangles*) and *vma2Δ***_***W***_**(*blue circles*) *versus* the concentation of Fe**^**III**^**citrate added to the growth medium.** Points are the averages of 2-3 independent batches; bars represent standard error. The *solid red line* is a simulation using Equation 1 and α_cyt_ = 0.185 h^−1^, α_vac_ = 0.176 h^−1^, and K_α_ = 15 mM. The *solid blue line* is the same except α_vac_ = 0.

To further gauge the vacuolar iron content of WT and *vma2Δ*_*W*_ cells, we cultured such cells in high (mM) concentrations of nutrient iron and monitored growth rates. As yeast lack a means of exporting iron, vacuolar iron storage typically provides resistance to excessive iron levels. WT cells grew rapidly until 20 to 30 mM Fe^III^ citrate had been added to the growth medium, beyond which the growth rate declined (Fig. 7, triangles). *vma2Δ*_*W*_ cells grew slower than WT in medium containing up to 10 mM iron and grew poorly in media containing higher iron concentrations (Fig. 7, circles). We suspect that under these high-iron conditions, *vma2Δ*_*W*_ cells were unable to sequester toxic iron in their vacuoles in a nontoxic form.

The shapes of the plots of Figure 6 suggested that we could simulate growth rates using Equation 1:

(1) $$\alpha =\alpha_{cyt}\left(\frac{1}{1+{\left(\frac{\left[Fe\right]}{K_{\alpha }}\right)}^{5}}\right)+\alpha_{vac}$$

which assumes two contributions to the growth rate, one that is sensitive to excessive iron in the media (α_cyt_) and another that is not (α_vac_). The observed experimental growth rate α was the slope of the ln(OD600) *versus* time in the exponential growth region. This empirical equation may only apply to the range of media iron concentrations investigated. It implies that excess cytosolic iron is toxic, whereas iron sequestered in the vacuole is not, as least in WT cells. The red line in Figure 7 simulates the growth rate of WT cells in which vacuoles sequester iron from the cytosol. The blue line simulates the growth of *vma2Δ*_*W*_ cells in which vacuoles are presumed unable to sequester iron in a benign form such as Fe^III^ polyphosphate.

*vma2Δ*_*W*_ cells grew more slowly than WT cells, even in media that was not supplemented with iron at high concentrations. Our MB studies indicate that these cells accumulated Fe^II^ ions. We considered that the Fe^II^ species in *vma2Δ*_*W*_ cells might be susceptible to Fenton chemistry and that this caused slow growth. We investigated this by treating two of the ^57^Fe-enriched samples used to generate spectra in Figure 2 with H_2_O_2_. In both experiments, the resulting MB spectra did not change noticeably – the NHHS Fe^II^ doublet continued to dominate (data not shown).

We also probed the effect of varying phosphate supplementation on the WT and *vma2Δ*_*W*_ strains, the latter of which contains little vacuolar polyphosphate (16). We grew both WT and *vma2Δ*_*W*_ cells in media containing low (0.5 mM) and high (21 mM) supplemented phosphate. This treatment did not have an effect on the oxidation state of iron in *vma2Δ*_*W*_ cells (Fig. S1). Both high- and low-phosphorus *vma2Δ*_*W*_ spectra were dominated by the Fe^II^ doublet. Curiously, the spectrum of high-phosphate cells exhibited a quadrupole doublet with parameters typical of Fe^III^ oxyhydroxide phosphate–associated nanoparticles. The same doublet was present in the low-phosphate spectrum, albeit at lower intensity. This result suggests that in *vma2Δ*_*W*_ cells, the formation of nanoparticles may have been limited by phosphate rather than iron. In contrast, the presence of high or low phosphate in the growth media had no noticeable effect on iron in WT cells.

**Figure 8:**
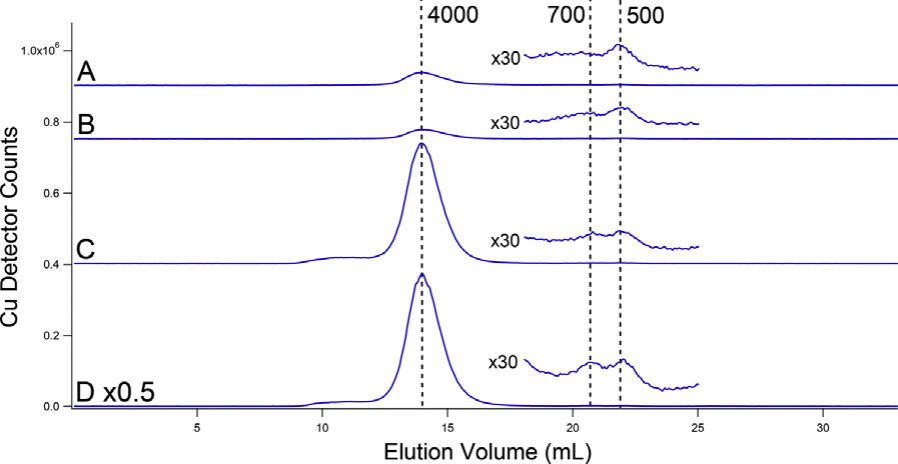
**Copper-detected chromatograms of cytosol FTS from two batches of WT (*A*–*B*) and *vma2Δ***_***W***_**(*C*–*D*) cells.** FTS, flow-through solution.

Although iron was our major focus, ICP-MS provides information on other elements, and so we also monitored Cu, Mn, Zn, S, and P. Copper was especially interesting because vacuoles also function in copper homeostasis. FTS from *vma2Δ*_*W*_ cytosol exhibited an intense copper-detected peak with an apparent mass of 4000 Da (Fig. 8). This peak was tentatively assigned to metallothionein Cup1 (38). The greater intensity of this peak in *vma2Δ*_*W*_ cytosol FTS relative to WT suggested greater expression of Cup1 and/or more extensive copper binding to this protein in *vma2Δ*_*W*_ cells. This makes sense because *vma2Δ*_*W*_ cells contained 2.5× more copper than WT cells (Table 1) and Cup1 sequesters copper. The same trend was reflected in the copper concentration of isolated cytosol (Table 1). The lack of a Cu^II^-based EPR signal in *vma2Δ*_*W*_ cells is consistent with diamagnetic Cu^I^ binding to metallothioneins. Also present were ca. 2 low-intensity Cu peaks in the range of 500 to 700 Da; their intensities were similar for both *vma2Δ*_*W*_ and WT FTSs (Fig. 8, insets).

**Figure 9:**
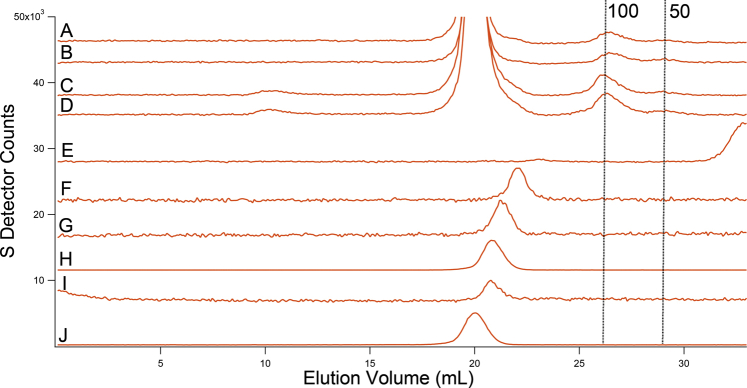
**Sulfur-detected chromatograms of cytosol FTSs and standards**. *A*–*B*, two batches of WT cytosol FTS. *C*–*D*, two batches of *vma2Δ* cytosol FTS. *E*–*J*, standards with areas normalized to indicate peak position. *E*, Na_2_S, *F*, cysteine, *G*, methionine, *H*, reduced glutathione, *I*, MgSO_4_, *J*, oxidized glutathione. The intense peak at ca. 20 ml in *A*–*D* is due to MES buffer (10 mM). FTS, flow-through solution.

LMM sulfur–based LC peaks were also detected in cytosolic FTSs from both WT and *vma2Δ*_*W*_ cells (Fig. 9). The dominant sulfur peak in the FTS from both cells (*V*_*e*_ = 20 ml) originated from the sulfur-containing MES buffer used early on in isolating the cytosol. The right shoulder of this peak coeluted with numerous sulfur standards including but not limited to cysteine (Fig. 9*F*). In *vma2Δ*_*W*_ cytosol FTS, the intensity of the shoulder was greater relative to that of WT FTS. The sulfur peak at an apparent mass of 100 Da. originated from the PMSF added to the buffer during isolation; the sulfur peak at an apparent mass of 50 Da originated from the DTT used earlier during isolation.

Hughes *et al.* (26) recently suggested that increased concentrations of cytosolic cysteine in *vma2*-mutant cells inhibited the vacuole-to-mitochondria iron trafficking pathway. To examine this, we grew *vma2Δ*_*W*_ cells on fermenting MM (pH 5) supplemented with 1 mM cysteine. The MB spectra of such cells were unaffected by cysteine supplementation (Fig. 3*B*) except for a modest decline in the CD (22% in the spectrum of unsupplemented *vma2Δ*_*W*_ cells down to 14% for the same cells supplemented with cysteine). Hughes *et al.* (26) suggested that cysteine supplementation caused “bioavailable” iron to decline and nonvacuolar cysteine to accumulate. The MB spectrum of the cysteine-supplemented *vma2Δ*_*W*_ sample did not show an increase in Fe^III^ nanoparticles, a form of iron considered to be bio unavailable.

**Figure 10:**
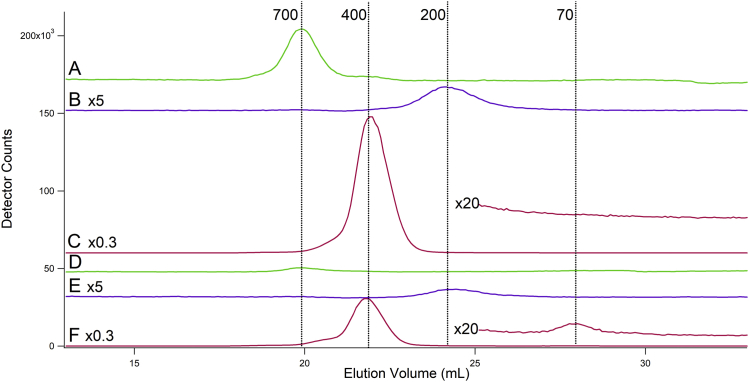
**Zn-, Mn-, and P-detected chromatograms of cytosol FTS from WT (*A*–*C*) and *vma2Δ***_***W***_**(*D*–*F*) cells.** Each displayed Zn (*A* and *D*), Mn (*B* and *E*), and P (*C* and *F*) trace is the average of two traces from independent batches. FTS, flow-through solution.

Mn and Zn chromatograms of *vma2Δ*_*W*_ and WT cytosol (Fig. 10, *A*–*B*) were like those described previously (38), including a single Zn peak at 700 Da and a single Mn peak at 200 Da. Phosphorus chromatograms (Fig. 10, *C–F*) exhibited a major peak at 400 Da and a shoulder at 600 Da. The intensities of Mn, Zn, and P peaks were significantly lower in *vma2Δ*_*W*_ traces, consistent with the lower concentration of these elements in mutant cells and isolated cytosol (Table 1). Notably, only the P peaks at 400 Da declined in intensity for *vma2Δ*_*W*_ cytosol; the peak at 600 Da remained the same. The peak at 70 Da comigrated with an AMP standard.

**Figure 11:**
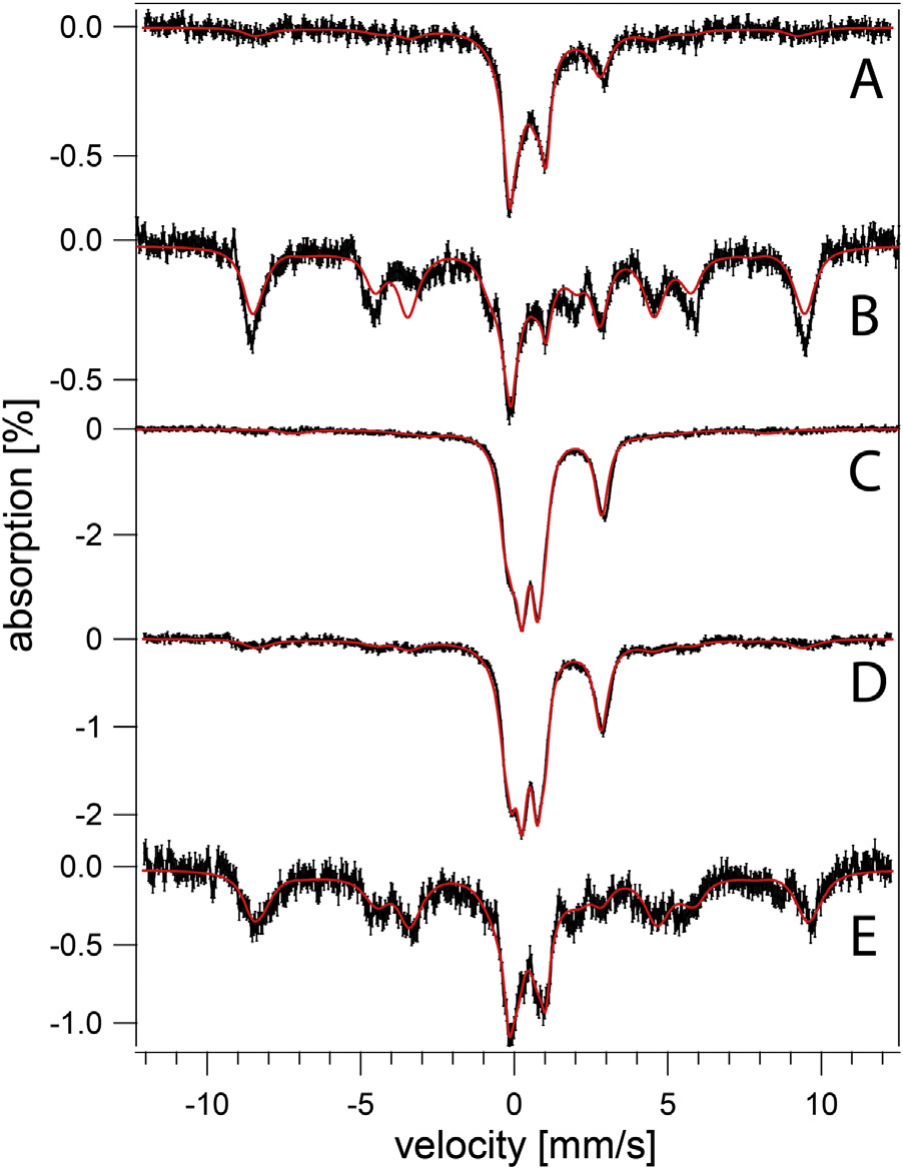
**Mössbauer spectra of fermenting BY4741 (*A*–*B*) and *vma2Δ***_***B***_**(*C*–*E*) cells grown in media buffered at the following pH values.***A*, 6; *B*, 3; *C*, 5; *D*, 4; *E*, 3.

We were puzzled that many of the results obtained using *vma2Δ*_*W*_ cells differed from published results on what was ostensibly the same mutant. However, the strain used to generate the mutant in those reports was BY4741, whereas ours was W303. We obtained the mutant in the BY4741 strain (to be called *vma2Δ*_*B*_) and collected MB spectra of those cells grown at different pHs in fermenting MM supplemented with 40 uM ^57^Fe citrate. *vma2Δ*_*B*_ cells grown at low pH exhibited spectra that were virtually indistinguishable from WT cells, including an intense magnetic feature due to vacuolar Fe^III^ (Fig. 11*E* *versus* Fig. 11*B*). At higher pH, *vma2Δ*_*B*_ cells exhibited spectra dominated by a broad quadrupole doublet due to Fe^III^ nanoparticles (Fig. 11*C–D*). Also, the percent absorbance was higher than in WT spectra, similar to that observed for ISC-mutant cells with an activated iron regulon (19, 20, 21). The spectra of *vma2Δ*_*B*_ cells grown at intermediate pH contained a stronger-than-WT NHHS Fe^II^ doublet (Fig. 11*D*), with parameters similar to those used to simulate the Fe^II^ doublet in *vma2Δ*_*W*_ cells.

**Figure 12:**
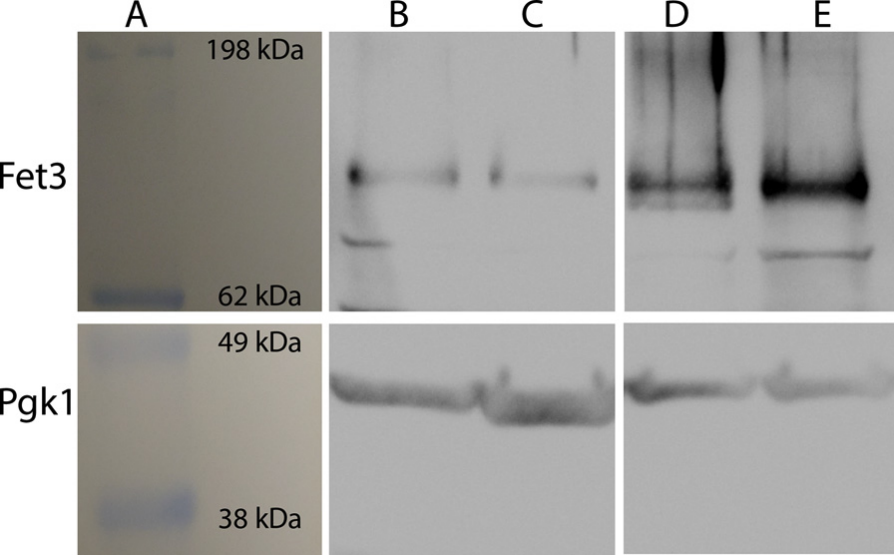
**Western blot of fermenting WT and *vma2Δ* whole-cell extract from both W303 and BY4741 background strains.***Top row*, anti-FET3. *Bottom row*, anti-PGK1. *A*, image of prestained protein ladder with masses indicated. *B*–*C*, w303 WT and *vma2Δ*_*w*_, respectively, cultured in MM supplemented with 40 μM iron. *D*–*E*, BY4741 WT and *vma2Δ_B_*, respectively, cultured in YPAD. All bands in each row were run on the same gel. MM, minimal medium, YPAD, yeast extract peptone adenine dextrose medium.

We attributed the differences between our results and those in the literature to differences between the W303 and BY4741 background strains. Notably, our experiments had not indicated an iron trafficking defect in the *vma2Δ*_*W*_ cells. Prompted by a reviewer’s comment, we sought to determine by Western blot of whole-cell extracts whether the iron regulon in *vma2Δ*_*W*_ cells was activated by monitoring the relative abundance of the high-affinity iron importer Fet3. Cytoplasmic Pgk1 was a positive control. We did not observe a significant difference in Fet3 abundance between *WT*_*W*_ W303 and *vma2Δ*_*W*_ cells (Fig. 12, top row, B and C respectively), indicating that the iron regulon is not activated. In contrast, Fet3 was more abundant in *vma2Δ*_*B*_ *versus* WT BY4741 cells (Fig. 12, top row, E *versus* D respectively), supporting previous studies indicating that the iron regulon is activated in *vma2Δ*_*B*_ cells grown in yeast extract peptone adenine dextrose medium (YPAD) media (22, 23).

## Discussion

As described in the Introduction, there is significant evidence for an iron trafficking pathway from vacuoles to mitochondria in yeast. Vacuoles store iron that is needed for mitochondriogenesis especially when cells shift from fermentation to respiration. Nonacidic vacuoles with defects in V-ATPase have been reported to generate mitochondrial defects in ISC synthesis and iron homeostasis. Our initial objective was to probe this relationship and defects in *vma2Δ*_*W*_ cells by MB and EPR spectroscopies and LC-ICP-MS chromatography. However, analyzing our results was more challenging than anticipated.

One result was clear: most of the iron in fermenting *vma2Δ*_*W*_ cells was nonheme high-spin Fe^II^. In W303 cells, approximately the same percentage of iron was HS Fe^III^, previously assigned to vacuolar iron. The overall iron concentrations in WT and *vma2Δ*_*W*_ cells and the spectral contributions of the CD (due to [Fe_4_S_4_]^2+^ clusters and LS Fe^II^ hemes) were also similar. We conclude that vacuolar Fe^III^ ions in WT cells are reduced to the Fe^II^ state in *vma2Δ*_*W*_ cells. We suspect but are not certain that the Fe^II^ remains in the organelle. We have observed a similar Fe^III^ → Fe^II^ reduction of vacuolar iron in yeast cells grown on adenine-deficient media in which metabolism has been altered (39). Some vacuolar iron in iron-replete respiring *vma2Δ*_*W*_ cells was reduced to Fe^II^ (and some may have formed Fe^III^ nanoparticles).

The reduction of Fe^III^ to Fe^II^ as the acidity of vacuoles declines can be partially or wholly explained by the conclusions of Raguzzi *et al.* (40) and Singh *et al.* (41) using insights from Schafer and Buettner (42). These researchers hypothesized that the glutathione redox couple $\left\{GSSG+2H^{+}+2e^{-}⇄2GSH\right\}$ controls the redox status of vacuoles in accordance with the Nernst equation:

$$E_{GSH}^{0\left(pH\right)}=E_{GSH}^{0\left(pH7\right)}-\frac{59}{2}\log\left(\frac{{\left[GSH\right]}^{2}}{\left[GSSG\right]{\left(\frac{\left[H^{+}\right]}{\left[10^{-7}\right]}\right)}^{2}}\right)$$

The standard half-cell reduction potential at pH 7 is *E*^*0(pH7)*^_*GSH*_ = −240 mV, whereas at pH 6, it is *E*^*0(pH6)*^_*GSH*_ = −180 mV (*i.e.*, more oxidizing under acidic conditions). On this basis, they correctly predicted that the iron in WT vacuoles should be Fe^III^. For each pH increase of 1 (a 10-fold drop in acidity), the effective reducing power of glutathione increases by 59 mV. Assuming pH 6 to 7 in *vma2Δ* vacuoles, the thermodynamic reduction potential for the Fe^III^/Fe^II^ couple in vacuoles should be in the range of the values listed above.

Interestingly, the same analysis predicts that the Fe^II^ should reoxidize to Fe^III^ in *vma2Δ*_*W*_ cells grown at lower pH, which was not observed; the dominant iron in these cells remained Fe^II^ for pH values as low as 3. Perhaps the pH of *vma2Δ* vacuoles is less sensitive to media pH than we have assumed. Other factors may also be involved (43). The redox state of vacuolar iron is likely influenced by the potential ligands in the organelle. For instance, vacuolar iron is coordinated by polyphosphate anions, and the concentration of these chains is diminished in *vma2Δ* vacuoles (16).

Diab and Kane predicted that *vma2Δ* cells should contain high levels of Fe^II^ ions (23). These researchers suggested that this form of iron is toxic to the cell because it participates in Fenton chemistry {Fe^II^ + H_2_O_2_ → Fe^III^ + OH^−^ + OH·}. However, we treated *vma2Δ*_*W*_ cells with H_2_O_2_ but found no evidence using MB spectroscopy that the Fe^II^ was altered in peroxide-treated cells. Reducing equivalents in the cell may have quickly reduced Fe^III^ back to Fe^II^ such that Fe^II^ remained dominant in the treated cells despite being used in Fenton chemistry. Upregulation of the cytosolic peroxidase Tsa2 in *vma2Δ* cells may have also diminished the effects of H_2_O_2_ (22).

Many of our results differed from expectations based on previous studies of *vma2Δ*_*B*_ cells. To examine whether there might be a strain dependence, we collected MB spectra of *vma2Δ*_*B*_ cells grown at different media pH values. Resulting spectra were closer to our expectations, with WT-looking vacuolar Fe^III^ in *vma2Δ*_*B*_ cells grown at low pH and NHHS Fe^II^ and nanoparticles for cells grown at high pH. BY4741 and W303 cells have different plasma membrane potentials and different sensitivity to alkali-metal salts (44). W303 cells are larger than BY4741 cells, their overall protein concentration is lower, and their vacuoles occupy a greater fractional cell volume (45). We found that *vma2Δ*_*W*_ cells were less sensitive to media pH and exhibited milder phenotypes associated with nonacidic vacuoles such that mitochondria were largely unaffected under the conditions used. We speculate that this reduced sensitivity might reflect larger vacuoles (a lower surface-to-volume ratio and therefore a smaller interface with the cytosol) in W303 cells, but further studies are required to verify this.

We have compared our results mainly to those of Kane and Hughes, but other studies report results regarding *vma* mutants that are more similar to ours. Ohya *et al.* (46) found that O_2_ consumption rates in *vma*-mutant cells (from parent strains other than W303 or BY4741) grown on glucose were “identical” to those of WT cells. They also reported no significant change in F-ATPase and succinate dehydrogenase activities, consistent with our results and with our suggestion of strain-dependent differences in *vma*-mutant phenotypes.

Hughes *et al.* (26) suggested that cytosolic accumulation of cysteine drives the low iron and oxidative stress response in V-ATPase–deficient cells. We observed a LMM sulfur species in isolated cytosol from WT and *vma2Δ*_*W*_ cells and found that its intensity increased in *Vma2Δ_w_* FTS relative to that of traces of WT FTSs. This shoulder to the MES buffer peak approximately comigrated with cysteine and other LMM sulfur species, including GSH and methionine. The increased shoulder intensity in *vma2Δ*_*W*_ isolated cytosol is consistent with the findings of Hughes *et al.*, though further confirmation of elevated cytosolic cysteine levels is required.

*vma2Δ*_*W*_ cells grew slower than WT cells and were more easily intoxicated by high concentrations of iron in the medium. We simulated these differences by assuming that *vma2Δ*_*W*_ cells are unable to sequester iron (or unable to sequester it in a benign form). However, the situation is undoubtedly more complicated. The toxicity of imported iron may also be associated with the oxidation state of that iron, with Fe^II^ being more toxic than Fe^III^.

*vma2Δ*_*W*_ cells and cytosol contained less Mn than in WT cells and cytosol; a similar decline has been observed in PPN1-UP cells (47) which have elevated levels of the polyphosphatase Ppn1. In both strains, cells contain low levels of polyphosphate relative to WT. Mn binds vacuolar polyphosphate (12), suggesting that these mutant cells may have responded to a decreased capability to store Mn in vacuoles by slowing Mn import.

The decline of phosphorus in *vma2Δ*_*W*_ cells and cytosol may be related to changes in the activity/expression levels of Pho84 and Pma1, both of which are located on the plasma membrane (14). Pho84 is a symporter that imports protons and phosphate ions into the cytosol; Pma1 exports protons from the cytosol. The activity of Pma1 decreases in *vma* mutants (7), which along with the loss of V-ATPase activity increases the acidity of the cytosol. We speculate that this might decrease the activity of Pho84 and thus reduce the import of phosphate into *vma* cells. Pho84 may also be involved in manganese and zinc import (48), and a decline in activity could have contributed to the observed decline of Mn and Zn concentrations in *vma2Δ*_*W*_ cells.

The increased concentration of Cu in our *vma2Δ*_*W*_ cells and cytosol confirms earlier reports that *vma*-mutant cells contain high levels of copper and that copper is dysregulated in these cells (17, 24). A similar accumulation may be driving the reported decline in Ctr1 expression in *vma2Δ*_*B*_ cells (22). Most LMM Cu in both WT and *vma2Δ*_*W*_ cytosol is probably bound to metallothionein Cup1. Higher copper concentrations in *vma2Δ*_*W*_ cells likely afforded increased levels of Cu-bound Cup1. The inability of nonacidic vacuoles to sequester copper may have increased the concentration of cytosolic Cu, which in turn induced expression of *CUP1*. Another possibility is that Cu-bound Cup1 is mobilized to vacuoles for degradation and that this process is inhibited in nonacidic vacuoles, thereby causing a buildup of Cu-bound Cup1 in the cytosol. A similar peak was tentatively assigned to Cup1 in chromatograms of vacuolar lysates (12).

Does iron trafficking in *Saccharomyces cerevisiae* include a direct or dedicated vacuole-to-mitochondria pathway? A direct pathway would involve contact of the two organelles (kiss-and-run) and would be independent of cytosol. A dedicated pathway would involve the synchronized export of a particular iron species from the vacuole into the cytosol, and as a second step, the import of that species exclusively into mitochondria. If the iron exported from vacuoles entered a cytosolic pool of iron that had various trafficking destinations, including but not limited to mitochondria, we would not regard that pathway as being direct or dedicated.

We have not systematically distinguished these possibilities, but our results disfavor direct or dedicated pathways. The vacuoles are nearly empty in cells grown in iron-deficient or iron-limited media (*e.g.*, 1 μM ^57^Fe citrate), yet MB spectra of such *vma2Δ*_*W*_ cells afforded a CD as intense as for equivalent WT cells. This suggests that vacuoles are unnecessary for iron to enter mitochondria and be used to generate ISCs. We tentatively conclude, pending further investigations, that cytosolic iron can enter mitochondria directly without first entering and exiting vacuoles and that there is no direct/dedicated vacuole-to-mitochondria trafficking pathway as defined above.

So how does the nonacidity of vacuoles negatively impact ISC metabolism in mitochondria? We suggest, following Hughes (26), that the effect indirectly involves vacuoles and directly involves the cytosol. Cysteine or a derivative thereof may be blocked from entering nonacidic vacuoles, and its buildup in the cytosol may inhibit iron traffic into mitochondria. Likewise, a hyperacidic cytosol, caused by the absence of V-ATPase to pump cytosolic protons into vacuoles and/or a decline in Pma1 activity, may also hinder iron trafficking. In support of this, we observed a shift in the LMM iron species in *vma2Δ*_*W*_ cytosol, from dominance of a species with an apparent mass of 600 Da to one with an apparent mass of 800 Da.

Finally, we have had difficulty throughout this study reconciling the milder phenotype of the *vma2Δ*_*W*_ strain with the more dramatic phenotype of *vma2Δ*_*B*_. Both mutant cells grow slowly and with difficulty under respiring conditions, but *vma2Δ*_*W*_ mitochondria are indistinguishable (by our methods) from WT mitochondria. In contrast, *vma2Δ*_*B*_ mitochondria show difficulty generating ISCs, the iron regulon is clearly activated, and nanoparticles and ROS are likely generated. Those cells recover only under conditions in which the media is supplemented with excessive iron.

We suggest that *vma2Δ*_*W*_ mitochondria suffer from the same originating problem as *vma2Δ*_*B*_ but to a milder degree. We hypothesize that the fundamental problem is a slow rate of iron import from the cytosol to mitochondria, due to the hyperacidity of this cellular region and/or to the inhibitory effect of high cysteine concentrations. The effect of slow iron import into mitochondria on the growth of *vma2Δ*_*W*_ cells would not be evident from our studies because we patiently waited for them to grow to sufficient absorbance at 600 nm prior to harvesting them for MB spectroscopy. By doing this, the ultimate iron content is expected to be like that of WT cells (apart from the reduction of vacuolar Fe^III^ → Fe^II^). For *vma2Δ*_*B*_ cells, the rate of iron traffic from the cytosol to the mitochondria might be so slow that the respiratory shield weakens and a vicious cycle ensues, leading to nanoparticles, ROS, and an activated iron regulon, similar to that observed in Mrs3/4ΔΔ cells, which can only import iron slowly into mitochondria under iron-limited growth conditions (49, 50). In *vma2Δ*_*W*_ cells, this effect might be muted.

## Experimental procedures

### Generation of the *vma2Δ* strain

Studies were conducted with the haploid strain of *S. cerevisiae*, W303 (MAT *α ade2-1, his3-3-11, 15 leu2-3,112, trp1-1, ura3-1*). The *VMA2* open reading frame was deleted in W303 by the Longtine method (51). Primers were synthesized including *VMA2* gene specific sequences (lower case) and plasmid specific sequences (upper case): F1 (agagtagacagtacatcaagcgaaaataaatattgcagga/CGGATCCCCGGGTTAATTAA); R1 (gacaaaataaaaaaagcctttttcttcagcaaccgtcctc/GAATTCGAGCTCGTTTAAAC).

A PCR was performed on template plasmid pF6A-His3MX6 (51) carrying the *S. pombe his5+* gene, and a 1403-bp DNA fragment was amplified as expected. The DNA product (about 5 μg) was used to transform W303 yeast using the lithium acetate procedure (52), and several colonies were selected for histidine prototrophy and carried forward. The correctness of the *VMA2* deletion was verified as follows. Genomic DNA was extracted from the transformants, and a PCR, using forward primer I (ctcatgaccgatggtacg) and reverse primer N (ATGTGATGTGAGAACTGTATC), was performed. The I primer resides in the 5’ region of *VMA2* gene outside the region of recombination. The N primer resides in the *TEF* terminator region of the pF6A-His3MX6 plasmid. The derived PCR product was approximately 688 bp in size and was present in the knockouts but not in the controls (Fig. S2), confirming that the *VMA2* flanking region and *His3MX6* module including the *S. pombe* *his5+* gene were correctly juxtaposed in the genome of the putative knockouts. The knockout strains had the expected auxotrophies and prototrophies and grew more slowly than the parent on rich or defined media.

### Cell growth

W303 WT (*MAT α ade2-1 his3-11,15 leu2-3,112 trp1-1 ura3-1*) and W303 *vma2Δ* (*MAT α ade2-1 his3-11,14 leu2-3,112 trp 1-1 ura3-1 Δvma2::His3MX6*) cells were obtained from frozen 15% glycerol stocks on YPAD (2% (w/v) glucose, 1% (w/v) yeast extract, 2% (w/v) peptone, 0.01% (w/v) adenine, 2% (w/v) bactoagar) plates. BY4741 WT (*MATa his3Δ1 leu2Δ0 met15Δ0 ura3Δ0*) and congenic BY4741 (*∆vma2::KanMX6*) cells were maintained similarly.

Individual colonies were inoculated in precultures of MM (20 g/l glucose, 5 g/l ammonium sulfate, 1.7 g/l yeast nitrogen base without ammonium sulfate and ferric chloride (MP Bio), 100 mg/l leucine, 50 mg/l adenine, 20 mg/l histidine, 20 mg/l uracil, 48 mg/l tryptophan, 10 μM copper sulfate) and either 1 or 40 μM ferric citrate. For two batches, the medium was supplemented with 1 mM cysteine. Media were buffered with a 0.05 M citric acid/sodium citrate buffer in varying ratios. For pH 3, 4, 5, 6, and 7, the citric acid-to-sodium citrate ratio was 13.3:1, 1.9:1, 1:1.4, 1:4.4, and 0:1, respectively. Minor adjustments were made with NaOH to achieve the desired media pH, as monitored using an Accumet AB15 pH meter.

BY4741 cells for Western Blot were cultured in YPAD media (20 g/l glucose, 10 g/l yeast extract, 20 g/l peptone, 100 mg/l adenine). Cell cultures were incubated at 30 °C and 180 rpm shaker speed. Absorbance was monitored at 600 nm using a Genesys6 spectrophotometer (Thermofisher). Fifty to 100 ml of precultures was transferred to 1- to 2-l media when absorbance at 600 nm was 0.7 ± 0.1. Cells were harvested from 1 to 2 l when culture was at exponential phase (absorbance at 600 nm = 0.8 ± 0.2). Cells were pelleted *via* centrifugation at 5000*g* for 5 min using a Sorvall LYNX6000 superspeed centrifuge (Thermofisher). Cells were washed twice with 1 mM EDTA and then twice with deionized water (5 ml of wash per gram wet cell pellet). After each wash, cells were pelleted as above and the supernatant was discarded. Finally, pellets were suspended in a minimal volume of double-distilled water and pelleted into MB cups by centrifugation at 6400 RPM for 10 min using a Beckman Coulter Optima L-90K ultracentrifuge (SW32Ti rotor); samples were frozen in liquid N_2_ for later analysis. EPR samples were pelleted after washing by centrifugation at 5000*g* for 5 min into EPR tubes.

For H_2_O_2_ studies, whole-cell MB samples were thawed and incubated in 1 ml 5% H_2_O_2_ for 15 min or in 200 μl of 0.0034% H_2_O_2_ (initial concentrations) overnight. Cell pellets were reacquired for MB spectroscopy *via* centrifugation at 5000*g* for 5 min.

### Respiring cell growth

Cell cultures were initially grown in 50 mL of fermenting MM as above. Once absorbance at 600 nm reached 0.6, the cells were pelleted and resuspended in 1.00 L of respiring MM (same as fermenting MM pH 5 except that 3% (v/v) glycerol and 1.5% (v/v) ethanol replaced glucose). Cells were harvested as described above.

### Varying phosphate cell growth

Cell cultures were grown in fermenting CSM media at 30 °C and 180 RPM. CSM media consisted of 20 g/l glucose, 1.6 g/l CSM, 5.6 g/l yeast nitrogen base minus phosphates (MP Bio), 100 mg/l NaCl, 60 mg/l adenine, 100 mg/l leucine, 48 mg/l tryptophan, 10 μM copper sulfate, and 40 μM ferric citrate. The medium was supplemented with 0.5 and 21 mM monopotassium phosphate. All such studies were conducted at pH 5 using a 0.05 M citric acid/sodium citrate buffer. Cells were harvested as above.

### Mössbauer and EPR spectroscopies

Mössbauer spectra were collected at 5 K and 0.05 T on a MS4 WRC spectrometer (SEE Co, Minneapolis, MN) calibrated at RT using an α-iron foil. The applied field was parallel to the gamma radiation. EPR spectra were collected in an X-band Elexsys EPR spectrometer (Bruker) using the following conditions: 9.3789 GHz microwave frequency, 25 dB attenuation, 0.6325 mW power, 100 kHz modulation, 10G modulation amplitude, 5000G sweep width, 120 s sweep time, 5 to 10 scans.

### Mitochondria studies

Mitochondria were isolated anaerobically from cells harvested from 24 L of fermenting cell cultures (pH 5), as described (35). Mitochondrial protein concentration was determined using the BCA assay. Mitochondria were pelleted *via* centrifugation at 12,000*g* for 10 min.

Aconitase was assayed as described (53, 54) but with minor changes. Mitochondria were handled within an anaerobic glove box (MBraun Labmaster 130) containing c.a. 5 ppm O_2_. A pellet of mitochondria containing 80 mg of protein was diluted to 250 μl with lysis buffer (degassed 4.7 mM Triton X-100, 50 mM NaCl, and 50 mM Tris-HCl). Two hundred microliters of the resulting solution was mixed with 200 μl of 50 mM NaCl, 50 mM Tris-HCl, and 50 μl of double-distilled water in a 1-mm pathlength quartz cuvette. The sample was sealed with a rubber septum and removed from the box. Fifty microliters of 10 mM *cis*-aconitic acid (Sigma-Aldrich) was added through the septum, and the sample was mixed and immediately monitored at 240 nm for 360 s using a Hitachi U-3310 spectrophotometer. The molar absorptivity of *cis*-aconitic acid at 240 nm (ε_240_) was 4.88 mM^−1^ cm^−1^ (54).

### Cytosol and vacuole isolation

The cytosol was isolated from fermenting cells (pH 5) and filtered through a 10-kDa cutoff membrane as described (55). Filtered FTS was injected into a treated size-exclusion (SEC) Superdex Peptide 10/300 Gl column (GE Life Sciences) connected to an Agilent 1260 Bioinert quaternary pump (G5611A) and Agilent 7700x ICP-MS. The mobile phase was 20 mM ammonium acetate pH 6.5 flowing at 0.6 ml/min. Vacuoles were isolated as described (12).

### Western blot

Western blot was completed using the whole-cell extract as described (12, 55). An anti-Fet3 antibody from rabbit was used in a 1:1000 dilution. The antibody was generated as described (28).

### Elemental analysis

Isolated cytosol samples (100 μl) or double-distilled water–washed whole cells (ca. 100 mg) suspended in fresh double-distilled water were digested with 500 μl of undiluted (70%) trace metal–grade HNO_3_ (Fisher) at 70 °C for ca. 16 h in plastic falcon tubes sealed using electrical tape. Samples were cooled to RT, and 250 μl of 35% hydrogen peroxide was added. Samples were resealed and incubated at 65 °C for 90 min. Samples were diluted to 10 ml with double-distilled water, affording c.a 3.5% and 0.86% (w/w) final concentrations of trace metal–grade nitric acid and hydrogen peroxide, respectively. A cell pellet packing efficiency of 0.7 and whole-cell pellet density of 1.1029 g/ml (56) were used in calculations.

## Data availability

All data except those described as “Data not shown” in the text are contained within the manuscript and SI.

## Conflict of interest

The authors declare that they have no conflicts of interest with the contents of this article.
