# Supplementary material for: Mössbauer and LC-ICP-MS investigation of iron trafficking between vacuoles and mitochondria in vma2ΔSaccharomyces cerevisiae
Source: J Biol Chem. 2020 Dec 6;296:100141. doi: 10.1074/jbc.RA120.015907 (PMC7948489; doi:10.1074/jbc.RA120.015907)

**Supplemental Material for Kim et al. 2020**

**Table S1. Percentage contribution of Mӧssbauer spectral features.** Percentages have estimated uncertainties of ± 4%. Non-heme high spin (NHHS) S = 5/2 Fe^III^ was simulated using parameters E/D = 0.3, ΔE_Q_ = 0.4 ± 0.1 mm/s, η = 2 ± 1, A_0_/g_n_β_n_ = -233 ± 4 kG, δ = 0.59 ± 0.04 mm/s, Γ = 0.70 ± 0.06 mm/s. Low spin (LS) hemes and S = 0 [Fe_4_S_4_]^2+^ clusters were simulated using parameters δ = 0.44 ± 0.2 mm/s, ΔE_Q_ = 1.17 ± 0.07 mm/s, Γ = 0.4 ± 0.1 mms/s. NHHS Fe^II^ was simulated using δ = 1.27 ± 0.02 mm/s, ΔE_Q_ = 2.9 ± 0.1 mm/s, and Γ = 0.6 ± 0.1 mm/s. Fe^III^ nanoparticles were simulated using δ = 0.52 mm/s, ΔE_Q_ = 0.65 mm/s, Γ = 0.6 ± 0.1 mm/s. F = fermenting, R = respiring, C = whole cell; M = mitochondria. Nutrient Fe^III^ citrate concentrations are in μM.

| **sample** | **pH** | **Figure** | **type ([Fe])** | **LS hemes and Fe_4_S_4_ clusters** | **NHHS Fe^II^** | **Fe^III^ nanoparticles** | **Non-heme HS Fe^III^** |
| --- | --- | --- | --- | --- | --- | --- | --- |
| WT_W_ | 7 | 1A | F, C (40) | 30 | 29 | 12 | 29 |
| WT_W_ | 6 | 1B | F, C (40) | 21 | 20 | 7 | 52 |
| WT_W_ | 5 | 1C | F, C (40) | 15 | 17 | 10 | 58 |
| WT_W_ | 4 | 1D | F, C (40) | 12 | 16 | 10 | 62 |
| WT_W_ | 3 | 1E | F, C (40) | 17 | 19 | 0 | 64 |
| *vma2Δ_W_* | 5 | 3A | F, C (40) | 17 | 56 | 27 | N/A |
| *vma2Δ_W_* + cys | 5 | 3B | F, C (40) | 14 | 64 | 22 | N/A |
| *vma2Δ_W_* | 4 | 3C | F, C (40) | 25 | 58 | 17 | N/A |
| *vma2Δ_W_* | 3 | 3D | F, C (40) | 22 | 55 | 23 | N/A |
| WT_W_ | 5 | 5A, B | F, M (40) | 71±12 | 18±1 | 11±11 | N/A |
| *vma2Δ_W_* | 5 | 5C, D | F, M (40) | 45±0 | 29±1 | 26±1 | N/A |
| WT_W_ | 5 | 6A | R, C (1) | 32 | 25 | 15 | 28 |
| WT_W_ | 5 | 6B | R, C (40) | 22 | 11 | 5 | 62 |
| *vma2Δ_W_* | 5 | 6C | R, C (1) | 29 | 36 | 35 | N/A |
| *vma2Δ_W_* | 5 | 6D | R, C (40) | 9 | 33 | 58 | N/A |
| WT_B_ | 6 | 11A | F, C (40) | 21 | 23 | 33 | 23 |
| WT_B_ | 3 | 11B | F, C (40) | 4 | 16 | 9 | 71 |
| *vma2Δ_B_* | 5 | 11C | F, C (40) | 0 | 29 | 61 | 10 |
| *vma2Δ_B_* | 4 | 11D | F, C (40) | 1 | 28 | 52 | 19 |
| *vma2Δ_B_* | 3 | 11E | F, C (40) | 9 | 8 | 17 | 66 |

**Figure S1.** Mössbauer spectra of fermenting WT (A and B) and *vma2Δ_W_* whole cell samples (C and D) grown in phosphate-deficient MM supplemented with 21 mM (A and C) and 0.5 mM (B and D) monopotassium phosphate. Samples were also supplemented with 40 μM ferric citrate and 10 μM cupric sulfate.


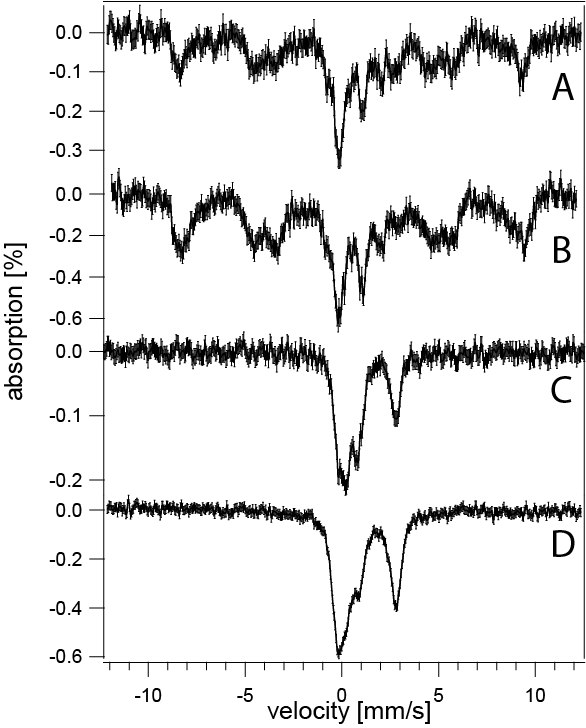


**Figure S2.** A confirmatory gel of the *vma2Δ_w_* construct. Genomic DNA was extracted from various yeast strains and used as a template for PCR using primers I from the *VMA2* genome and N from the plasmid *TEF* terminator region. Left to right: lane 1-4 W303 *∆vma2::his5* knockout candidates; lane 5, W303 wild-type; lane 6, BY4741 *∆vma2::kanR* constructed using pFA6a-kanMX6 in analogous fashion; lane 7, DNA markers from the 1 kb Fermentas ladder starting at 500, 750, 1000 bp. The presence of the 688 bp amplified fragment indicates the correctness of the knockout and the juxtaposition of the *VMA2* genomic piece next to the *his5+* containing plasmid sequences.


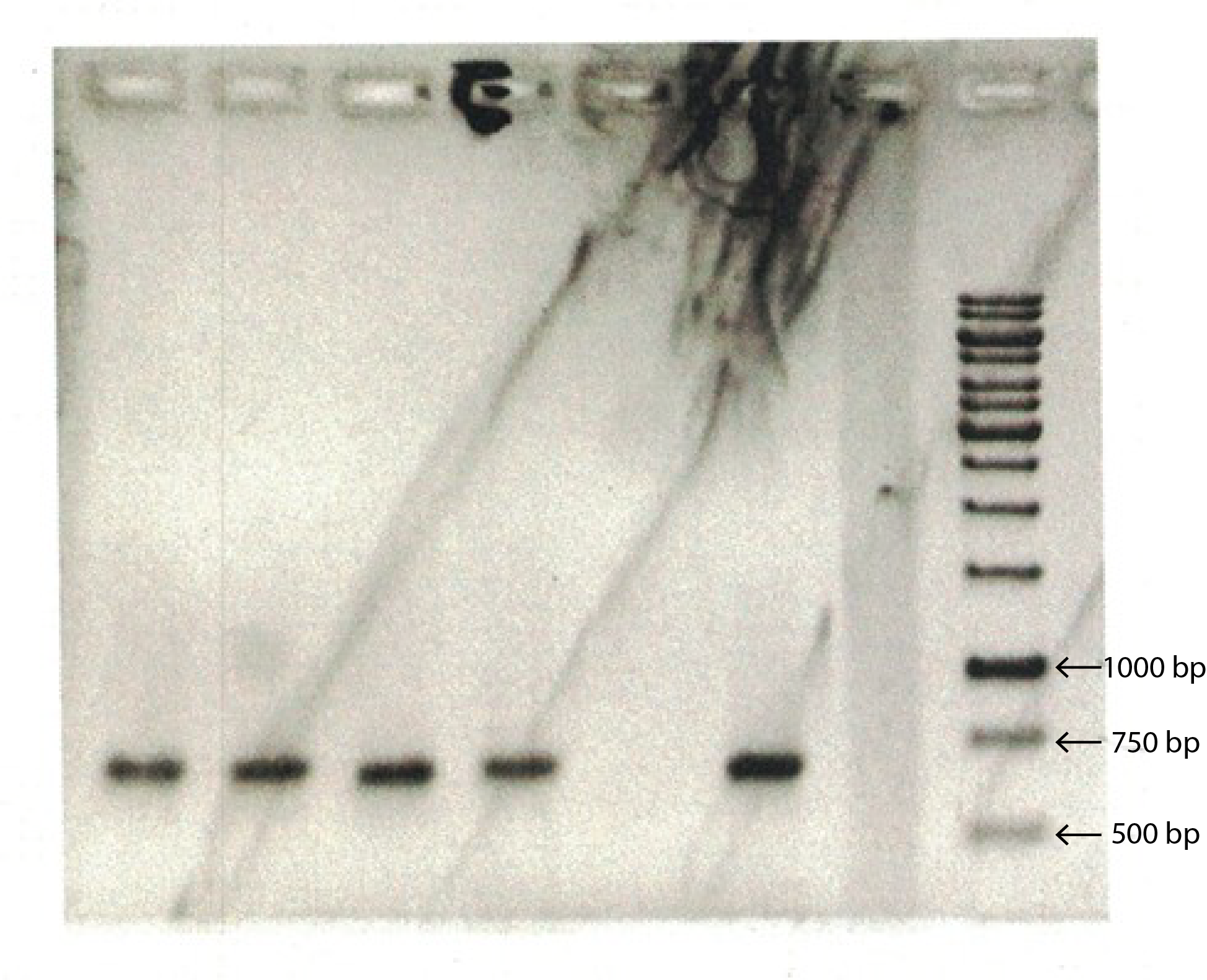

Supplement: Supplementary Tables and Figures [file mmc1.docx]
